# Detection of *Cyclospora* in captive chimpanzees and macaques by a quantitative PCR-based mutation scanning approach

**DOI:** 10.1186/s13071-015-0872-8

**Published:** 2015-05-15

**Authors:** Marianna Marangi, Anson V Koehler, Sergio A Zanzani, Maria T Manfredi, Emanuele Brianti, Annunziata Giangaspero, Robin B Gasser

**Affiliations:** Department of Science of Agriculture, Food and Environment, University of Foggia, Foggia, Italy; Faculty of Veterinary and Agricultural Sciences, The University of Melbourne, Victoria, Australia; Department of Animal Pathology, Hygiene and Public Health, University of Milan, Milan, Italy; Department of Veterinary Sciences, University of Messina, Messina, Italy

**Keywords:** *Cyclospora*, *q*PCR, Mutation scanning-based sequencing, Non-human primates, Zoonosis

## Abstract

**Background:**

*Cyclospora* is a protistan parasite that causes enteritis in several species of animals including humans. The aim of this study was to investigate the presence of *Cyclospora* in captive non-human primates.

**Methods:**

A total of 119 faecal samples from *Pan troglodytes*, *Macaca sylvanus, Cercopithecus cephus*, *Erythrocebus patas*, *Chlorocebus aethiops* and *Macaca fascicularis* from a wildlife animal rescue center as well as from *Macaca fascicularis* from an experimental primate research center were tested for the presence of *Cyclospora* by quantitative real-time PCR (*q*PCR) and single-strand conformation polymorphism (SSCP) analysis.

**Results:**

*Cyclospora* was detected in three *Pan troglodytes* (13.6%) and nine (9.3%) *Macaca fascicularis*.

**Conclusions:**

The present study represents the first record of *Cyclospora* in captive primates in Europe, suggesting the presence of *Cyclospora cayetanensis*, which is transmissible to humans.

## Background

Enteritis caused by protistan parasites is a principal cause of substantial morbidity in humans, often resulting in mortality in under-privileged communities [1]. Protozoa belonging to the genus *Cyclospora* (Apicomplexa: Eimeriidae) are obligate, intracellular parasites that infect the epithelium of the intestines or bile ducts of a variety of hosts, mostly vertebrates [2]. Nineteen species of *Cyclospora* have been described in millipedes, reptiles, insectivores, rodents, and human and non-human primates [2]. In humans, *Cyclospora* was first identified in Papua New Guinea in the late 1970s [3] and then named *Cyclospora cayetanensis* [4,5] in the early 1990s. *Cyclospora cayetanensis* has since been recognized as an important cause of endemic or epidemic diarrhoeal illness in children and immuno-compromised or -suppressed individuals worldwide [6]. Human cyclosporosis is endemic in developing countries, but also in industrialized settings, mainly in people with no history of foreign travel [7] and sometimes implicated in community-wide outbreaks of diarrhoeal illness [7].

*Cyclospora* species appear to be underestimated as infectious agents, and very little is known about their epidemiology (including host and geographical ranges) and pathogenicity [7,8]. Infections are transmitted via the faecal-oral route through contaminated environmental water, food or soil, although oocysts need to sporulate outside of the mammalian host (from 7–15 days) to be infective [9].

Investigations of outbreaks and case–control studies have shown that people with cyclosporosis had contact with animals, suggesting a zoonotic role of *C. cayetanensis. Cyclospora* oocysts, morphologically resembling *C. cayetanensis*, have been detected in chickens, ducks, cattle, mice, rats and dogs [10-14], whereas morphologically and molecularly characterized *C. cayetanensis* have been identified in dogs, domestic birds and in monkeys (*Mulatta mulatta*) [10]. This evidence supports the possible zoonotic nature of *C. cayetanensis*, although whether animals from which this parasite have been isolated are natural hosts is still unknown.

Investigations of *Cyclospora* of animals, particularly those with a close genetic relationship with humans, have important implications for better understanding the epidemiology and host range of members of this genus. PCR-based tools employing various nuclear (e.g., ribosomal and heat shock protein) gene markers have been utilized to detect and/or identify *Cyclospora* spp. [15]. In the present study, a quantitative PCR (*q*PCR), combined with single-strand conformation polymorphism (SSCP)-based sequencing analysis, was used for the detection and genetic characterization of *Cyclospora* from various species of captive primates using the second internal transcribed spacer (ITS-2) of nuclear ribosomal DNA as a marker.

## Methods

### Samples and isolation of genomic DNA

Fresh faecal samples from 119 captive, non-human primate individuals were collected in Italy; 22 samples were from *Pan troglodytes*, *Macaca sylvanus, Cercopithecus cephus, Erythrocebus patas*, *Chlorocebus aethiops* and *Macaca fascicularis* from a Wildlife Animal Rescue Center (WARC; 2008) and 97 from *M. fascicularis* from an Experimental Primate Research Center (EPRS; 2011–2012) (Table 1). Primates were kept in enclosures strictly according to the Guidelines for the Care and Use of Laboratory Animals of the Ministry of Health, Italy. None of the animals studied showed clinical signs, such as diarrhoea, at the time of sampling. Faecal samples (packed in individual plastic bags) were sent frozen to the Parasitology Laboratory, Department of Agriculture Science, Food and Environment, University of Foggia, Italy, where they were stored at −80°C. Subsequently, samples were thawed, and genomic DNAs were isolated from individual faecal samples using the Qiagen stool kit (Macherey-Nagel, Germany), according to the manufacturer’s instructions. DNA was eluted in 50 μl of H_2_0, quantified using a Qubit 2.0 fluorometer and stored at −20°C. The individual genomic DNA samples contained 0.2 to 100 ng per μl.

**Table 1:** **Number and species of investigated non-human primates and samples test-positive for** ***Cyclospora*** **by both** ***q*** **PCR and SSCP analysis**

**Collection sites**	**Animal number**	**Species**	**Common name**	**Sex** ^**a)**^	***Cyclospora***		**No. of oocysts** ^**b)**^
					***q*** **PCR**	**SSCP**	
WARC	1	*Pan troglodytes*	Chimpanzee	f	**+**	**+**	498
	2	*Pan troglodytes*	Chimpanzee	m	-	-	0
	3	*Pan troglodytes*	Chimpanzee	m	**+**	**+**	420
	4-6	*Pan troglodytes*	Chimpanzee	m	-	-	0
	7	*Pan troglodytes*	Chimpanzee	f	**+**	**+**	482
	8-12	*Pan troglodytes*	Chimpanzee	f	-	-	0
	13-15	*Pan troglodytes*	Chimpanzee	f	-	-	0
	16-18	*Macaca sylvanus*	Barbary macaque	m	**-**	**-**	0
	19	*Chlorocebus aethiops*	Green monkey	m	-	-	0
	20	*Macaca fascicularis*	Cynomolgus monkey	f	-	-	0
	21	*Erythrocebus patas*	Patas monkey	m	-	-	0
	22	*Cercopithecus cephus*	Moustached monkey	m	-	-	0
EPRC	1	*Macaca fascicularis*	Cynomolgus monkey	f	**+**	**+**	314
	2	*Macaca fascicularis*	Cynomolgus monkey	f	**+**	**+**	470
	3	*Macaca fascicularis*	Cynomolgus monkey	f	**+**	**+**	439
	4	*Macaca fascicularis*	Cynomolgus monkey	f	**+**	**+**	89
	5-55	*Macaca fascicularis*	Cynomolgus monkey	f, m	**-**	**-**	0
	56	*Macaca fascicularis*	Cynomolgus monkey	m	+	+	285
	57	*Macaca fascicularis*	Cynomolgus monkey	m	+	+	196
	58	*Macaca fascicularis*	Cynomolgus monkey	m	+	+	162
	59	*Macaca fascicularis*	Cynomolgus monkey	m	+	+	232
	60	*Macaca fascicularis*	Cynomolgus monkey	m	+	+	80
	61-97	*Macaca fascicularis*	Cynomolgus monkey	m	-	-	0

WARC: Wildlife Animal Rescue Center; EPRC: Experimental Primate Research Center.

^a)^f = female; m = male.

^b)^Per gram of feaces.

### Quantitative *q*PCR and melting curve analysis

The *q*PCR assay and melting curve analysis were performed in a CFX-96 Real Time Instrument (BioRad, Italy) [16]. Briefly, PCR was carried out in a final volume of 20 μl, utilizing SsoFast™ EvaGreen® Supermix (cat. no. 172–5201; Bio-Rad, Italy) and 0.5 μM of each primer CCITS2-F (forward: 5′-GCAGTCACAGGAGGCATATATCC-3′) and CCITS2-R (reverse: 5′-ATGAGAGACCTCACAGCCAAAC-3′) to a region (116 bp) within ITS-2 of *C. cayetanensis* (cf. [17]). Genomic DNA (50 to 100 ng), cloned ITS-2 (0.5 pg; reference, positive-control) DNA or water (negative control) in 5 μl were added to the reaction. Cycling conditions were: initial denaturation at 98°C for 2 min, followed by 35 cycles at 98°C for 5 s, and 59°C for 15 s. Fluorescence data were collected at the end of each cycle as a single acquisition. Melting curve analysis was performed at the end of each PCR run (70°C to 95°C at 0.5°C/5 s). Each sample was analyzed in duplicate, and the amplification cycle threshold (*Ct*) and melting temperature (*Tm)* values were calculated. The diagnostic *Tm* peak for *Cyclospora* was 84.5°C. The criteria used to define a test-positive sample were: (a) a detectable amplification curve, (b) a *Tm* value of ± 0.5°C with reference to the *Tm* value of plasmid control, and (c) a dF/dT fluorescence value of > 2.

Raw data were normalized by applying curve-scaling to a line of best fit, so that the highest fluorescence value was 100 and the lowest was zero (standard normalized melt curve). Then, the curves were differentiated, and a composite median curve was constructed using the median fluorescence values for each sample. The melting traces for each sample were subtracted from this composite median curve to draw a residual plot (difference graph). The number of DNA copies per μl was calculated by relating the *Ct* mean value of each sample to a standard curve for the plasmid control, and the number of oocysts calculated, assuming that an oocyst contains 15 copies of rDNA [18].

### *q*PCR performance

The analytical sensitivity of the assay was estimated using 10-fold serial dilutions (from 10^10^ to 10 copies/μl) of the (cloned) reference (positive) control, which were each subjected (in triplicate) to *q*PCR and subsequent melting curve analysis, and the mean value of the threshold cycle (*Ct*) was plotted against the logarithm of DNA copies per μl. The standard curve was produced by a linear regression of the plotted points and the range of linearity and the lowest detectable amount of DNA were estimated. PCR efficiency (*E*) was calculated according to the equation: *E* = 10^−1^/slope – 1 [19]. *E* of between 90 and 110% and a correlation (R^2^) of < 1 value were considered acceptable. To determine repeatability, three standard points of (cloned) reference (positive) control (10^7^, 10^5^ and 10 copies/μl) were tested in triplicate three times in the same experiment and the *Ct* mean values were recorded. The same standards were tested once a day for three more days to determine reproducibility. To analyze the reproducibility and repeatability, both intra- and inter-assay coefficients of variation (*CV*) were assessed. *CV* mean values were calculated following the formula: CV = σ*(Ct)*/μ *(Ct).*

### Single-strand conformation polymorphism (SSCP) analysis and sequencing

All amplicons were subjected to single-strand conformation polymorphism (SSCP) analysis using protocol B [20]. Amplicons representing distinct banding profiles were selected and treated with exonuclease I and shrimp alkaline phosphatase (Fermentas), according to the manufacturer’s instructions, and then subjected to direct, automated sequencing (BigDye Terminator v.3.1 chemistry, Applied Biosystems, USA) using the primer CCITS2-R. The quality of each sequence was assessed based on the corresponding electropherogram using the program BioEdit, and the sequences determined were compared with known reference sequences using the Basic Local Alignment Search Tool (BLAST; http://www.ncbi.nlm.nih.gov/BLAST).

## Results and discussion

Upon assessing assay performance, we established that intra-assay *CV* values for the 10^7^, 10^5^ and 10 standard points were 1.8%, 1.5%, and 1.2%, and inter-assay *CV* values for the 10^7^, respectively 10^5^ and 10 standard points were 0.80%, 0.60%, and 0.93%, respectively. The range of linearity was acceptable from 10^10^ to 10 copies/μl, with an amplification efficiency (*E)* of 93.6% and a slope of −3,486 (R^2^ = 0.995). The *q*PCR was then applied to the test samples.

Twelve of 119 (10.1%) faecal samples were *q*PCR test-positive for *Cyclospora*, including 3 (13.6%) chimpanzees (*Pan troglodytes*) from 22 primates from the WARC and 9 (9.3%) from 97 cynomolgus monkeys (*syn.* crab-eating macaque) (*Macaca fascicularis*) from the EPRC (Table 1). The numbers of *Cyclospora* oocysts in test-positive samples were predicted to range from 80 to 498 per gram of faeces (Table 1). All 12 *Cyclospora* ITS-2 amplicons produced in *q*PCR were subjected to SSCP analysis, and one profile was displayed for both non-human primate species. The sequence representing all 12 amplicons was identical to the reference sequence with GenBank accession no. AF301386.

Although the sequence of these amplicons was consistent with those determined for *C. cayetanensis*, some caution is needed in assigning species status, for the following reasons: (*i*) it is not yet known whether ITS-2 provides unequivocal specific identification of *Cyclospora* and (*ii)* we did not detect oocysts of *Cyclospora* for morphological identification/description. Therefore, although *Cyclospora* DNA was amplified using primers designed to ITS-2 of *C. cayetanensis* [17], the magnitude of sequence variation within individual, recognized species of *Cyclospora* as well as the levels of difference among recognised species of *Cyclospora* have not yet been rigorously assessed.

Although various non-human primates have been reported to harbour unique *Cyclospora* species, such as *Cyclospora cercopitheci* n. sp. in green monkeys, *C. colobi* n. sp. in colobus monkeys, and *C. papionis* sp. n. in baboons [21-23], the finding of *Cyclospora-*like oocysts in chimpanzees (*P. troglodytes*), baboons (*Papio cynochephalus*) [11] and in drills (*Mandrillus leucophaeus*) [14] raises the vexed question as to the specific identity of these parasites and whether they are transmissible to humans or not.

Given that attempts to infect different animal species (chickens, ducks, mice, gerbils, hamsters, rabbits, rats, sand rats, ferrets, pigs, dogs, monkeys and baboons) with *C. cayetanensis* have been unsuccessful [24], it is possible that *Cyclospora* species are predominantly host-specific, like *Eimeria* [25]; thus, further investigation is warranted in this regard. The identification of *Cyclospora* in a chimpanzee supports the previous detection of this parasite in this primate species [11]; if *C. cayetanesis* is confirmed, the role of *M. fascicularis* as a reservoir of this protozoan might support its molecular detection in a related species of macaque (i.e., *Macaca mulatta*) in Nepal [10].

While coprophagia is a likely mode of transmission of parasitic infections in captive primates [26], the animals studied here might have become infected by *Cyclospora* through water, soil or food (mostly fruit). Indeed, contaminated tap water and fresh produce (fruits and leafy vegetables) [27-29] are recognized as key sources of cyclosporosis outbreaks in humans [7,30], while the role of contaminated soil has only been suspected [8,29] but not yet proven.

It is possible that cynomolgus monkeys from the EPRS premises had been infected by *Cyclospora* upon arrival from China, where *Cyclospora*-like organisms have been detected recently in golden snub-nosed monkeys (*Rhinopithecus roxellanae*) from the Breeding Research Center in Shaanxi province, China [23], and where cyclosporosis in humans has often been reported [31-33]. Regarding chimpanzees from WARC, it is possible that the contamination might have been acquired locally, since the animals were not recently moved or imported from other areas. In Italy, humans have been found to harbour *C. cayetanensis* [34-36], and this parasite has also been isolated from stored tap water [37].

## Conclusions

Although the possible zoonotic role of *Cyclospora* detected herein cannot be confirmed, the high number of *Cyclospora* oocysts shed in faeces might be a public health concern, considering their resilience in the environment and to routine chemicals used to disinfect water [7,38], which might facilitate their dissemination *via* water and food and the environment to humans and/or other animals. Understanding the host and distributional ranges of *Cyclospora* species and transmission thereof should have important implications for preventing and controlling cyclosporosis. Whether the non-human primates studied here serve as natural reservoirs for *C. cayetanensis* remains unclear, and future investigations are warranted to confirm the specific status of *Cyclospora* detected in the present study.

## Abbreviations

*q*PCR: quantitative real-time PCR
SSCP: Single-strand conformation polymorphism
